# Tailoring cultural offers to meet the needs of older people during uncertain times: a rapid realist review

**DOI:** 10.1186/s12916-022-02464-4

**Published:** 2022-08-24

**Authors:** Stephanie Tierney, Sebastien Libert, Jordan Gorenberg, Geoff Wong, Amadea Turk, Kerryn Husk, Helen J. Chatterjee, Kathryn Eccles, Caroline Potter, Emma Webster, Beth McDougall, Harriet Warburton, Lucy Shaw, Nia Roberts, Kamal R. Mahtani

**Affiliations:** 1 University of Oxford, Oxford, Oxfordshire UK; 2 University of Plymouth, Plymouth, UK; 3 University College London, London, UK

**Keywords:** COVID-19, Cultural sector, Older people, Realist review, Social prescribing, Well-being

## Abstract

**Background:**

Non-medical issues (e.g. loneliness, financial concerns, housing problems) can shape how people feel physically and psychologically. This has been emphasised during the Covid-19 pandemic, especially for older people. Social prescribing is proposed as a means of addressing non-medical issues, which can include drawing on support offered by the cultural sector.

**Method:**

A rapid realist review was conducted to explore how the cultural sector (in particular public/curated gardens, libraries and museums), as part of social prescribing, can support the holistic well-being of older people under conditions imposed by the pandemic. An initial programme theory was developed from our existing knowledge and discussions with cultural sector staff. It informed searches on databases and within the grey literature for relevant documents, which were screened against the review’s inclusion criteria. Data were extracted from these documents to develop context-mechanism-outcome configurations (CMOCs). We used the CMOCs to refine our initial programme theory.

**Results:**

Data were extracted from 42 documents. CMOCs developed from these documents highlighted the importance of tailoring—shaping support available through the cultural sector to the needs and expectations of older people—through messaging, matching, monitoring and partnerships. Tailoring can help to secure benefits that older people may derive from engaging with a cultural offer—being distracted (absorbed in an activity) or psychologically held, making connections or transforming through self-growth. We explored the idea of tailoring in more detail by considering it in relation to Social Exchange Theory.

**Conclusions:**

Tailoring cultural offers to the variety of conditions and circumstances encountered in later life, and to changes in social circumstances (e.g. a global pandemic), is central to social prescribing for older people involving the cultural sector. Adaptations should be directed towards achieving key benefits for older people who have reported feeling lonely, anxious and unwell during the pandemic and recovery from it.

**Supplementary Information:**

The online version contains supplementary material available at 10.1186/s12916-022-02464-4.

## Background

‘Non-medical’ issues affecting health and well-being, such as loneliness or lack of purpose, are matters of concern for older adults [1, 2]. Health problems associated with ageing can have a negative impact on confidence [3], bringing unique challenges in sustaining social relations for older people [4]. The Covid-19 pandemic amplified social isolation experienced by older people (who were identified as being at greater risk from complications of the virus if they contracted it) due to restrictions on in-person interaction [5, 6].

Social prescribing enables healthcare professionals to refer patients to community-based support and activities [7]. It is one way to address issues of well-being in later life during and beyond the Covid-19 pandemic. Social prescribing is an important mode of intervention in countries like the UK [8–10], the USA [11, 12], Australia [13] and Canada [14]. It relies on ‘link workers’ (they may be known by other titles, including ‘community connectors’ and ‘social prescribers’ [15]) who connect people to social or community-based services and activities that can help with their non-medical issues.

Part of the rationale for employing link workers is to improve patients’ holistic well-being and, by doing so, reduce demands on healthcare staff, particularly general practitioners [16]. In England, the NHS is funding link worker posts in primary care [17]; patients are often referred to a link worker by their general practitioner. Link workers have time to talk to a patient to find out what is of concern to them and what would help to improve their current situation. These employees should have a good knowledge of local services, organisations, charities and activities, which could help with a range of non-medical issues.

Among the range of community services that link workers might connect individuals to, cultural institutions (such as public gardens, libraries and museums) are promising for social prescribing [18, 19]. The closure of these venues during the pandemic reduced their capacity to support the community. However, the cultural sector has been resilient, setting up alternative means to reach people through digital and remote activities [20, 21].

We were funded to conduct a programme of research by the UK Research and Innovation (UKRI) Arts and Humanities Research Council; as part of this work we completed a rapid realist review. It aimed to understand how, for whom, in what ways and why the cultural sector, as part of social prescribing, can improve the well-being of older people (aged 60 years and above) in the context of the Covid-19 pandemic. We refer to ‘cultural offers’ below, meaning activities or spaces (online or in person) provided by a cultural organisation. This might include (but is not limited to) guided walks around a botanical garden, book groups run by a library or volunteering at a museum.

## Methods

Realist reviews are an appropriate way of explaining how complex interventions (such as social prescribing) work, for whom, in what circumstances and why [22]. They are underpinned by a realist philosophy of science, which entails thinking about generative causation—the notion that outcomes are produced by mechanisms (often hidden), which may or may not be triggered depending on context [23]. Within a realist review, literature is drawn upon to develop explanations that focus on mechanisms that produce outcomes, and contexts required to trigger these mechanisms. It involves the development of context-mechanism-outcome configurations (CMOCs). CMOCs inform and are embedded within a programme theory—a proposition of how an intervention works, for whom, under what conditions and why [24].

We conducted a rapid realist review, defined as a “time responsive” approach to developing policy-sensitive recommendations on a topic [25]. A rapid, or restricted, approach has become popular across systematic review types; they are restricted in terms of truncating elements of the process [26]. In our review, this included using a limited number of databases to locate relevant papers (CINAHL, EMBASE, MEDLINE, Cochrane databases). We also sought to identify grey literature on the Repository for Arts and Health Resources (www.artshealthresources.org.uk) and were sent relevant documents by experts in the field.

**Fig. 1:**
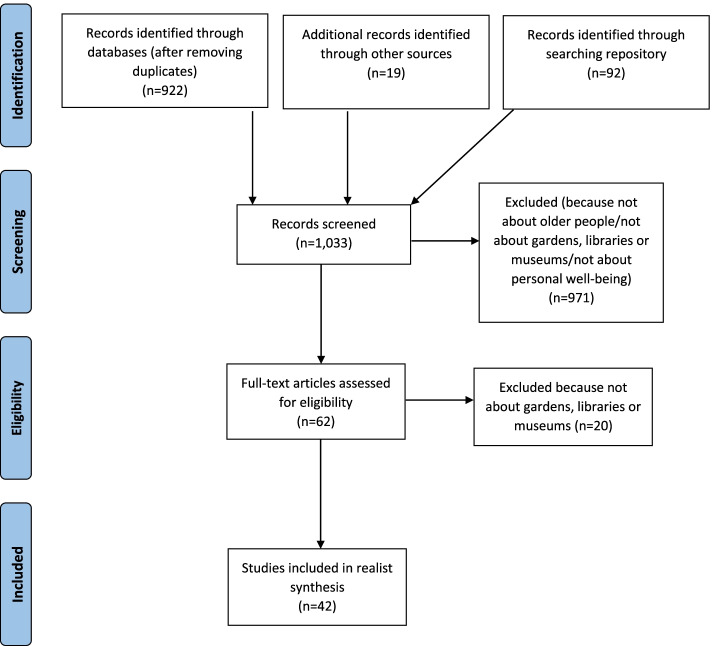
Searches and screening for the review

Figure 1 shows the flow of references from screening to inclusion. Searching for and screening of documents ran between October 2020 and January 2021. Methods we used to identify papers and the review’s eligibility criteria have been published in a blog (https://socialprescribing.phc.ox.ac.uk/news-views/views/locating-data-sources-for-a-rapid-realist-review-on-the-cultural-sector-and-social-prescribing-for-older-people). We followed the RAMESES (Realist And Meta-narrative Evidence Syntheses: Evolving Standards) reporting guidelines for realist reviews [27]. As it was a rapid review, we focused on identifying literature related to curated/public gardens, libraries and museums. However, feedback we received from a range of stakeholders whilst producing the review (see below) suggests that our findings relate to other cultural areas and activities.

We started the project by creating an initial programme theory in the form of a diagram, which we have published elsewhere [28]. Our team comprises expertise on social prescribing across disciplines and sectors, and this first iteration was based on our existing knowledge of social prescribing [19] and discussions with representatives from the cultural sector. It proposed how the cultural sector, through social prescribing, might work for older people, under what conditions. Through our engagement with the literature during the rapid realist review, we tested and refined the initial programme theory by considering how emerging CMOCs changed or extended our understanding of the topic.

In line with a realist logic, the analysis explored connections between context, mechanism and outcome to explain how cultural institutions (curated/public gardens, libraries and museums) might play a role in the well-being of older people as part of social prescribing, especially within the context of the Covid-19 pandemic. The qualitative computer programme NVIVO was used to organise data and identify key concepts; it has been noted as a useful tool for data management and analysis in a realist project [29]. Two reviewers (JG and SL) extracted data from included documents into broad concepts. They coded included literature within NVIVO, using this programme to help with clustering sections across documents that were on a similar topic. They initially coded based on broad terms (e.g. importance of space/place, sociability, cultural sector adaptability, patient expectations, link worker understanding). They used these broad concepts to think about CMOCs. They developed and shared their thinking with the rest of the research team in the form of written narratives. We also discussed emerging CMOCs with our project partners (representatives from the cultural sector and those involved in social prescribing) and our public involvement group (composed of older people) in the form of short presentations. Written notes were taken at these meetings. CMOCs were then amended based on this feedback.

Papers were assessed in terms of being ‘fit for purpose’ based on their ability to contribute to programme theory development [30]. This called for researchers to consider whether they contained useful data for extending or testing the emerging CMOCs and/or programme theory (‘relevance’) and examining, when necessary, whether the piece of data used was underpinned by credible and trustworthy methods (‘rigour’). We enhanced the rigour of our work through regular meetings with our project partners and our public involvement group. In February 2021, we also held three stakeholder meetings via Zoom, which were attended by older people, cultural sector staff and link workers (n=40 in total). At these meetings, we shared early findings from the review, which people discussed in small groups. CMOCs presented below came mainly from the reviewed literature but were augmented through conversations we had during these stakeholder meetings.

As is expected for this type of synthesis, we progressively focussed our realist review [27]. Our findings centre more on understanding the engagement of older people with cultural offers (i.e. the earlier parts of our initial programme theory). We judged this focus was important to understand initially because without engaging, any benefits from social prescribing for older people are not going to occur. Furthermore, the literature we found was mostly focussed on engagement.

## Results

**Fig. 2:**
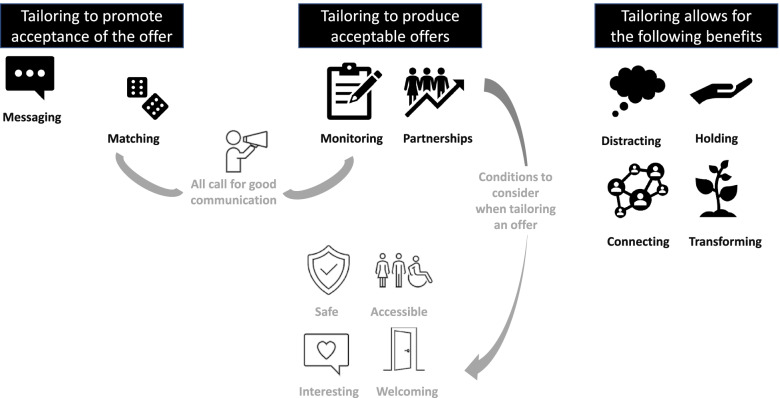
Programme theory. Our rapid realist review highlighted how tailoring can help to ensure that an older person is receptive to the idea of engaging with a cultural offer. It also suggested some broad aspects of tailoring that cultural providers should consider when developing cultural offers (e.g. making sure people feel safe—physically and psychologically, that they can access offers—physically but also online, creating offers that are entertaining or absorbing, and providing a welcoming atmosphere, whether in-person or online). Through tailoring to optimise uptake of cultural offers and enjoyment/engagement with them, we propose that older people may experience one or more of the following benefits—being distracted from worries/concerns, feeling psychologically held, connecting with others, transforming their sense of self and place in the world

After screening 1033 references, data were extracted from 31 scientific articles and 11 reports [20, 21, 31–70] (details of included documents can be found in Additional file 1). Through our analysis, we created 18 CMOCs, which were used to refine our initial programme theory. These were discussed as a team and resulted in the development of a key concept included in the revised programme theory (see Fig. 2)—‘tailoring’. In broad terms, tailoring can be seen as the shaping of an intervention and its distribution to meet the needs of recipients (and providers) in a way that reflects the environment and circumstances in which it is delivered. In the review, tailoring relates to responses from link workers and cultural sector staff to older people’s needs, preferences and priorities, and to changing social situations (including a global pandemic). Tailoring underpins the pathway that enables older people to obtain benefits from a cultural offer as part of social prescribing, which we summarise in Fig. 2. These benefits can arise from the space in which the offer is provided (e.g. a building), from interactions involved or activities undertaken. In the rest of this section, we describe components of tailoring and the benefits that older people can receive from cultural offers.

### Components of tailoring

Through our engagement with the included evidence, and discussions with stakeholders, we identified and developed the following components of tailoring. The CMOCs that underpin and explain these components of tailoring are in Table 1.

**Table 1:** CMOCs that underpin components of tailoring in the connection of older people to the cultural sector as part of social prescribing (see Additional file 2 for supporting data)

**Messaging**	
* • CMOC1: When a link worker can provide detailed information about a cultural offer (C), the older person is more likely to understand if it is suitable for them (O) because they can work out what it entails (M).*	
* • CMOC2: When the link worker explains the cultural offer as part of social prescribing in a way that shows how it relates to an individual’s needs (C) because it is regarded as a credible solution (M), the older person is more likely to accept it (O).*	
**Matching**	
* • CMOC3: When link workers understand the needs and expectations of an older person (C), they are more likely to suggest a suitable cultural offer (O) because they have an understanding of what is acceptable to and needed by that individual (M).*	
* • CMOC4: When a link worker has information of local social prescribing options (C), they can match these to older people's needs and expectations (O) because they have the necessary knowledge (M).*	
**Monitoring**	
* • CMOC5: If cultural institutions evaluate the cultural offers they make to older people (C), they can adapt the suitability of the offer (O) because they are aware of the changes needed (M).*	
* • CMOC6: When a link worker asks for feedback from older people attending cultural offers (C), they can assess whether a cultural offer is benefiting someone (M) and changes can be made to the individual’s action plan if required (O).*	
* • CMOC7: When link workers and cultural sector staff collaborate constructively (C), improvements to cultural offers are more likely (O) because their shared knowledge is harnessed (M).*	
**Partnerships**	
* • CMOC8: When a cultural organisation is committed to supporting public well-being (C), because staff feel that they are undertaking such work in a facilitative environment (M) they are willing to make changes and take risks (O).*	
* • CMOC9: When older people are consulted about the content of cultural offers (C), something is developed by cultural organisations that is appropriate and acceptable to end users (O) because it has taken into consideration their ideas (M).*	
* • CMOC10: When link workers and cultural sector staff interact (C), it allows for greater understanding and valuing of each party’s contribution to older people’s well-being (M), which promotes a willingness to collaborate (O).*	

#### Messaging—the means through which cultural offers as part of social prescribing are communicated (CMOCs 1–2)

Tailoring highlights how any offer proposed as part of social prescribing must be considered suitable by the recipient. Messaging applies to how someone is connected to a cultural offer as part of social prescribing (e.g. through seeing a link worker). Simply providing information may not be sufficient for such a suggestion to be adopted. Older people may want further details about what a cultural activity would entail [44]. Confidence is a common issue affecting older people’s participation in cultural activities [58, 63]. Hence, reassurance about the safety and welcoming nature of an offer is important [39, 44]. Our consultations with stakeholders emphasised that receiving adequate information plays a role in influencing expectations and decisions to join a cultural offer as part of social prescribing. Acceptance will be influenced by the value given to and recognition of the cultural sector’s role in supporting well-being. This may be shaped by how the idea of a cultural offer is presented to an older person (e.g. as a credible, positive opportunity).

#### Matching—having a good insight of what an older person might be open to trying and might benefit from, and having appropriate offers to connect them to (CMOCs 3–4)

The variety of requirements and expectations that people have in later life means that understanding and addressing an individual’s needs within social prescribing are key [48, 66]. This is a task for link workers to undertake. As part of tailoring, link workers discuss with an older person what they want/need, which may affect what they are directed towards. For tailoring to ensue, link workers must develop a relationship with an older person so they know what an individual expects. They need to identify who might be receptive to a cultural offer and consider how they propose this suggestion so it sounds like a viable source of support (see messaging above). They also need to be aware of what cultural offers are available locally [45]. Having a variety of activities is useful, to cater for a range of needs [67].

#### Monitoring—checking that cultural offers are acceptable and adapting them, when necessary, based on feedback and input from stakeholders (CMOCs 5–7)

Reviewed literature referred to the integration of evaluation and monitoring when developing interventions [55, 60]. Similarly, stakeholders stated that designing systems to receive feedback from older people and link workers creates cultural offers that are appropriate and address the needs of those involved in social prescribing. A solid monitoring and evaluation scheme may be necessary to demonstrate to funders the benefits that can transpire from cultural sector activities for social prescribing [55]. For link workers, keeping abreast of available cultural offers and checking with older people if they meet their needs is also important.

#### Partnerships—as social prescribing centres on human interaction (even when delivered remotely), positive relationships among different parties are required (CMOCs 8–10)

Partnerships are essential to identifying the diversity of circumstances, needs and expectations in later life, and tailoring an offer accordingly. Stakeholders reported that cultural institutions may need support and insight from other agencies to grasp the complex combination of characteristics and circumstances experienced by older people, and/or may not know how to reach individuals in need. Collaborations are important in this regard. The reviewed literature provided relevant examples of strategic collaborations with third sector organisations [49, 67], healthcare providers and clinicians [44, 45] and care homes [58]. Stakeholder feedback stated that communication between link workers and cultural sector staff creates an understanding of the contribution each can make to the well-being of older people. It means that link workers can advise on key information they need when proposing a cultural offer to older people. Interaction between cultural sector staff and older people ensures that offers are acceptable to and appropriate for the latter. Partnerships within a cultural organisation are also required; if an organisation is committed to supporting public well-being, and is open to some risk-taking, it will permit change and enable innovation to flourish. Outreach work may form part of this innovation [55]. Innovation has been required during Covid-19, when buildings were closed and cultural offers had to be delivered remotely (online but also by post or telephone). The importance of taking into account the well-being of cultural sector staff has been noted, as delivering these activities can be demanding; one way to address this is to have several staff working together [67].

### Potential benefits for older people from a cultural offer

As we articulate in Fig. 2, it is through tailoring that benefits of cultural offers for older people can be realised. Some benefits may be quick to transpire but short lasting, others may be slower to emerge but more profound. For example, if an older person is seeking to escape from problems momentarily, then engaging with an exhibition online or in person may be appropriate. If they wish to make connections, they may need to attend several meetings or activities (in person or online).

Below, we explore the different benefits that can be derived from a cultural offer; the underpinning CMOCs are in Table 2.

**Table 2:** Benefits to older people from engaging with cultural offers as part of social prescribing (see Additional file 2 for supporting data)

**Distracting**	
* • CMOC11: When an older person finds the cultural offer stimulating (C), they experience an escape from their problems (O) because they enjoy and are absorbed by the activity (M).*	
* • CMCO12: When the cultural offer engages older people’s senses (C), their enjoyment increases (O) because their mind is elsewhere (M).*	
**Holding**	
* • CMOC13: When the cultural environment is older people friendly (C) they enjoy attending (O) because they feel safe and at ease (M).*	
* • CMOC14: When the cultural offer is delivered professionally and consistently (C), older people feel reassured (O) because they know what to expect (M).*	
**Connecting**	
* • CMOC15: When the cultural offer provides a social component (C), older people feel less lonely (O) because they have been facilitated to engage in human interactions (M).*	
* • CMOC16: As the cultural offer continues to provide a social component (C), older people can increase their social network (O) because they have been facilitated to develop and maintain new relationships (M).*	
**Transforming**	
* • CMOC17: When the cultural offer enables older people to experience or learn new things (C), their self-esteem and confidence increase (O) because they are encouraged to try things outside of their comfort zone (M).*	
* • CMOC18: When older people are given the option to take part in a cultural offer in a way that suits their preferences (C), their self-worth is increased (O) because they feel attended to (M).*	

#### Distracting—cultural offers can provide an immediate boost to people’s well-being (CMOCs 11–12)

Older people may welcome being distracted temporarily from worries by focusing on an offer and learning or trying something new [31, 32, 44, 63]. In the cultural sector, this can happen through stimulation, which can take a range of forms. For instance, handling objects can promote well-being through sensory engagement [39]. Similarly, online cultural offers can provide a multisensory experience by using 3D models of artworks that enable users to engage their imagination [42].

Offers can provide a break from daily routines, prompting people to do something different [36, 48, 58]. Having a consistent meeting time and location for a cultural offer may help with this [67], providing a structure to the week and giving people something to look forward to [48]. In terms of tailoring, link workers need to decipher how much structure an older person wants before referring them to a particular cultural offer.

#### Holding—cultural offers can afford people a sense of safety and belonging (CMOCs 13–14)

Cultural settings can be spaces where older people feel safe and included—in an ‘older person friendly’ environment. Professionalism and consistency of experience may be required for this to transpire. For example, knowing that staff will be warm and welcoming enables older people to relax and become immersed in a cultural offer [63]. When attending an offer for the first time, cultural sector staff can orient older people, to prevent them from feeling alone and to assuage any anxieties [58].

Efforts should be made to reduce stereotyping and stigmatisation in cultural settings [45], so that older people are treated respectfully as valued visitors [38, 44]. This includes providing adequate support for any physical or cognitive limitations, to prevent alienation. Co-production of cultural offers with older people may be important in this respect [55, 57].

Veall and colleagues [67] advised that cultural sector staff be mindful of a venue’s accessibility; awareness training around potential difficulties that older people may encounter when navigating a venue has been proposed, so that signs of distress are recognised and addressed [57]. Online offers can reduce the stress of traveling to a physical location [21]. Nevertheless, it is important to ensure that digital platforms are easy to navigate [21, 42, 65], and to assist people in their use, if necessary, so they maintain a sense of psychological safety or comfort.

Distress and discomfort can arise when a cultural offer is not suited to the needs and interests of older people. For instance, offers that involve lots of walking can be exhausting [62]. Viewing certain images or pieces of art could be unsettling and may unearth unpleasant memories [65, 67]; however, our consultations with stakeholders suggested this could be cathartic, if staff are available to help people manage how they feel. Offers that are tailored to the needs and interests of older people contribute to them feeling calm and welcomed [49].

#### Connecting—cultural offers can catalyse new social connections (CMOCs 15–16)

The literature identified making social connections as a vital element to cultural offers [36, 67, 68]. Participants in one article suggested socialisation should be a priority when designing an offer because they linked this to improved health and well-being [68]. This could be facilitated by having a shared point of focus within a cultural activity to promote dialogue between people who are strangers [40]. To make people feel at ease, having a facilitator experienced in knowing when and how to stimulate and moderate discussion is important [63].

Once the cultural offer ends, older people can maintain social connections by meeting on a more informal basis [62]; the cultural venue can act as a base to facilitate this [63]. Cultural offers enable older people to develop their communication skills by expanding the things they can discuss with others [63]. Yet not all cultural offers must contain a social element as not all older people will be seeking to make connections through social prescribing.

Online cultural offers may not necessarily replace face to face interactions [21] but they can provide an avenue for socialisation. They are a means of reaching a wider range of isolated/vulnerable people [42], although consideration is required to ensure the needs of individuals with sensory difficulties are addressed [20]. Online offers delivered through platforms with the capability for social interaction (e.g. Zoom) allow users to establish relationships, reducing isolation [21], with some research suggesting that as older people become accustomed to an online interface they enjoy it more as their confidence increases [42, 65]. To prevent fatigue from screen use, it is recommended that online sessions are kept short [21]. What is not clear is how short they should be; this is something that could be explored through co-producing cultural offers with older people.

#### Transforming—engaging with cultural offers can lead to self-growth and empowerment (CMOCs 17–18)

Engaging in the arts is associated with increased control and autonomy for older people [66]; cultural offers provide them with an opportunity to express themselves and to engage in pursuits that they perceive to be worthwhile [55]. Confidence, self-esteem and self-direction can be built [44, 66] by learning information or developing skills [32, 38], such as mastering how to use a digital application to access an online cultural offer [42, 65].

There was some evidence that connecting with others in a cultural setting can shape how older people see themselves. Todd and colleagues [63] reported that it enabled them to recognise their personal strengths, as they took steps to improve their situation by trying new things and then sharing their learning. This research also highlighted the positive reinforcement of self-worth and likability that older people received from communicating with peers at cultural settings.

Trying new things and receiving encouragement from staff and others can build self-confidence [63]. However, not all older people want to learn or try something new. Hence, outcomes for each person involved in social prescribing have to be tailored to their specific expectations and needs. Furthermore, although self-growth and transformation can transpire from engaging with a cultural offer, getting someone to accept an offer in the first place may be a problem due to a lack of self-confidence. This highlights the importance of tailoring through messaging, matching, monitoring and partnerships (see above) to encourage people to try something that may be unfamiliar.

### Substantive theory: Social Exchange Theory

We considered the programme theory (Fig. 2) and associated CMOCs (Tables 1 and 2) in relation to substantive theories. As we wanted to further our understanding of tailoring and its potential impact on actors (older people, link workers, cultural sector staff), we were most interested in theory that would help us to do this. Social Exchange Theory was regarded as appropriate in this regard.

Homans [71] described human interactions in terms of costs, rewards and reinforcement—core concepts of Social Exchange Theory. What is exchanged in a social interaction might not be explicit. Social rewards can be intrinsic (e.g. feeling valued, respected, understood) or extrinsic (e.g. goods, services). Costs vary and include (but are not limited to) time, energy and money. When someone regards a social exchange as inequitable, frustration can ensue if they feel they are losing more than they gain.

Ongoing exchanges that result in reciprocal benefits foster trust, create interdependence and reduce uncertainty [72, 73]. Relationships are likely to continue through mutual reinforcement and disrupted when someone experiences a lack of symmetry between rewards and costs. This could have implications for (a) older people’s response to a cultural offer as part of social prescribing, (b) interaction between the cultural sector and link workers and (c) the role of the cultural sector in social prescribing for older people.

### Social Exchange Theory and our review findings

Social Exchange Theory provides support from existing substantive theory for the key role of tailoring identified within the review—of what is offered, to whom and by whom. Social prescribing can be seen as an exchange between different actors. Our review shows how tailoring forms part of this, as summarised in Table 3, which provides an overview (not necessarily exhaustive) from the literature of potential rewards and costs associated with social exchange and tailoring of cultural offers for older people as part of social prescribing.

**Table 3:** Examples of potential rewards and costs for actors as part of a social exchange in relation to tailoring of cultural offers for older people through social prescribing

Actors	Rewards	Costs
*Older person*	• Being listened to and understood • Getting clear information about available cultural offers • Being referred to an appropriate cultural offer • Making connections with new people	• Trying things that do not improve their situation or that make them feel uncomfortable (e.g. if stigmatising or patronising) • Encountering difficulties with accessibility when trying a cultural offer
*Link worker*	• Having a wider range of offers to propose to older people • Feeling able to make a difference to an individual’s situation • Contributing to shaping a cultural sector offer for older people	• Time to find and learn about local cultural offers • Having their suggestions for improving a cultural offer ignored • Finding interactions with cultural sector staff or older people difficult
*Cultural sector staff*	• Being able to reach a wider audience; expanding the range of people using their service • Seeing the difference that a cultural offer can make to an individual’s life	• Time to develop a cultural offer that is appropriate and accessible • Time to connect with link workers • Receiving negative feedback from link workers or older people

Link workers have to invest time and energy into understanding an older person and their needs. They are often paid as an employee for this, but they may receive other benefits (e.g. seeing people they are helping improve their life situation through engaging with a cultural offer). Link workers and cultural sector staff may decide to interact based on anticipated costs and rewards. When an older person gains from a cultural offer, it can foster trust between a link worker and cultural offer provider, although there needs to be a system of communication between these parties so they know that an older person has benefitted from a cultural offer. For an older person to take up (and hopefully sustain) engagement in a cultural offer suggested by a link worker, it is important (at first) that they perceive rewards from the offer as matching or exceeding any costs. What an older person may provide in terms of this exchange includes feedback on a service or showing gratitude to a link worker or cultural sector staff. In terms of benefits for cultural sector providers from engaging in social prescribing, they may receive referrals from a link worker that could diversify the audience engaging with their organisation. They may also receive advice from link workers on how to improve what is provided so it reflects the needs of older people. They are likely to continue providing cultural offers as part of social prescribing if feedback they receive suggests they are making a difference to people’s lives. If they feel that costs associated with such work are not mitigated by the rewards, they may pull back from providing cultural offers.

The overarching concept of tailoring from our review and further understanding brought from Social Exchange Theory emphasise the potential for co-production in ensuring that, as far as possible, cultural offers are shaped to meet the needs and circumstances of older people. Co-production “means delivering public services in an equal and reciprocal relationship between professionals, people using services, their families and their neighbours. Where activities are co-produced in this way, both services and neighbourhoods become far more effective agents of change” [74]. Adopting this approach to service development and delivery can ensure that individuals are working towards a common goal, reforming and potentially improving what is offered. Using co-production has the additional benefit of reducing an older person’s perception that any social exchange they are taking part in is inequitable; it can help them to feel that they can contribute to the development of cultural offers and that their voice is being heard and their ideas valued. For link workers, co-production can broaden the options they have available as offers they are happy to refer people to as part of social prescribing.

Rewards from co-production for any actors must not be undermined by associated costs (some of which are listed in Table 3); this will shape whether all parties remain engaged. It has been noted that co-production is not risk free [75]. Tensions may arise due to the relinquishing of power, conflicting priorities and misunderstandings, alongside the potential need for additional resources and time [76, 77]. If costs outweigh rewards, co-production may not continue.

## Discussion

Later life encompasses a variety of needs and circumstances [48, 58] that can influence receptiveness to a cultural offer [55]. Health issues such as dementia or frailty [34, 44], or risk aversion and factors affecting confidence [35, 56, 63], may shape an older person’s response to a link worker’s suggestion that they consider a cultural offer. The pandemic is likely to have heighted these issues for some older people who, our consultations with stakeholders suggested, may worry about safety in venues, and may have experienced a drop in confidence due to social isolation and a disconnection from their usual social networks. We also know that the delivery of social prescribing has changed due to Covid-19, with more interaction between link workers and people they support being undertaken remotely (rather than face-to-face) [78, 79]. Link workers had to be agile in their response to the pandemic [80], as services and activities in the community to refer people on to closed or moved online. It also called for cultural providers to be creative in how they continued to engage with the public when buildings were closed [81–83]. The importance of cultural and creative activities in providing people with solace and escapism has been recognised by the Local Government Association [84] in England; it announced a commission to promote the value of cultural provision during and recovery from the pandemic.

Variability in how older people and cultural providers have responded to the pandemic, and how link workers undertake their role, emphasise the importance of tailoring, a key component of our programme theory. For example, link workers must understand an individual and their needs and have a good range of local options to propose to older people [85]. As noted in our programme theory, positive outcomes that may come to older people who engage with cultural offers include being distracted (absorbed), feeling held (e.g. safe and accepted), making connections and transforming through self-growth. In part, this depends on what people are seeking through a cultural offer, which can be explored with a link worker. For some older people, being in a calm or relaxing space may be most important, others may want to learn, whilst for some the social aspect will be key.

As part of tailoring, the cultural sector must be responsive to older people’s needs by addressing things like accessibility and social circumstances (e.g. Covid-19). There will be limits to what is possible in terms of tailoring a cultural offer due to resource constraints and having the time to make changes. It may be a case of tailoring what is essential, which can be decided through co-production of offers with older people. Guidelines for museum and healthcare professionals to tailor activities for older people have been produced [60]. A checklist has also been created of what the cultural sector needs to do to provide an age-friendly environment [57]. Tailoring in this way may help to increase inclusivity in terms of who makes use of cultural offers. Future research could look to creating and evaluating a tailoring checklist to assist cultural providers when thinking about essential things to be accommodated (e.g. transport, toilets, support to attend if lacking confidence). When tailoring is not possible, a cultural offer may only be used by those who would have engaged without being connected through a link worker. This fits with criticism from Weiner [69] that museums tend to work with “known and ‘safe’ communities.” Support from inside and outside an organisation may be required for this to change. Social prescribing could form part of this shift in audience composition.

Training may be helpful for staff if a cultural offer has a social component, so they can support people new to a group; this may include having a range of activities as part of an offer to suit the differing preferences of those present (e.g. listening and watching, discussions, creative tasks, working in pairs) [63]. In terms of training, the pandemic has highlighted that cultural sector staff may require support to deliver attractive and engaging online offers. Likewise, older people may need assisting in their use, and link workers in how to encourage those unfamiliar with digital platforms to try them. During the pandemic, tailoring was required in response to the closure of cultural sector buildings. Digital provision was produced and is likely to form part of delivery going forwards. It has been advised that online offers should be non-patronising and “work to the capabilities not to the limitations of older audiences” [55]. Co-production may assist with this.

### Strengths and limitations

Throughout the review, we engaged in conversations with key stakeholders from the cultural sector, social prescribing and with older people, to ensure that our findings resonated with these individuals. A realist approach enabled us to draw on a range of literature. At the point of searching for literature, we found few documents meeting our inclusion criteria that related specifically to the pandemic (due to its recentness). The role of link workers was also missing from most documents we reviewed, as were data on digital offers. We plan to explore these topics in more detail in future research, through primary data collection, using a mixture of qualitative and quantitative methods. This will enable the programme theory presented in this paper to be tested and amended to further understanding of this topic.

We adopted a rapid approach to the review. This meant that the literature search was curtailed, and although extensive (covering grey literature as well as articles in academic journals), it was not exhaustive. There was sufficient information to produce a number of CMOCs that helped us to develop a programme theory that resonated with stakeholders. However, there are elements of our initial broad research question that remain unanswered from our review, which we will seek to address within our planned primary research through a series of interviews with older people and cultural providers, and a questionnaire with link workers.

## Conclusions

Tailoring was identified as a key concept within this review to ensure that cultural offers as part of social prescribing meet the needs and aspirations of older people. This has been especially necessarily in light of restrictions imposed by the Covid-19 pandemic across the world. Tailoring can happen at a range of levels (e.g. cultural sector staff being agile and link workers needing to know what older people require and expect). Older people can help with generating appropriate or acceptable cultural offers through feedback and taking a role in co-production. Tailoring can help to ensure that older people benefit from cultural offers; benefits might include a temporary distraction from worries in life, being psychologically held (feeling safe and included), expanding their social network or changing their self-perception. How to tailor cultural offers so they create feelings of safety and connection is an area for further exploration, especially if these offers are to be delivered online as well as in-person. Our review provides some indication of how this might be achieved.

## Supplementary Information

**Additional file 1.** Overview of included papers.

**Additional file 2.** A selection of data extracts used to develop Context-Mechanism-Outcome Configurations (CMOCs), which were also informed by our discussions with stakeholders.

## Data Availability

The review drew on published documents that others can access. We have referenced papers included in the review, which others can access should they wish.
