# Supplementary material for: Tailoring cultural offers to meet the needs of older people during uncertain times: a rapid realist review
Source: BMC Med. 2022 Aug 24;20:260. doi: 10.1186/s12916-022-02464-4 (PMC9398500; doi:10.1186/s12916-022-02464-4)
Supplement: Supplementary file 1 — Additional file 1. Overview of included papers. [file 12916_2022_2464_MOESM1_ESM.docx]

***Additional file 1: Overview of included papers***

| Number in reference list of main paper | Author(s), date and country | Aim of study | Type of study or document | Sample | Data collection and analysis | Type of venue(s) |
| --- | --- | --- | --- | --- | --- | --- |
| 31 | Age UK (2018)  United Kingdom | To delve further into findings around creative and cultural participation – what it is, who does what, and how it differs depending on people’s  overall level of wellbeing | Report | N/A | N/A | Cultural sector (museum) |
| 32 | Ander et al. (2013)  United Kingdom | To investigate the impact of museum object handling sessions on hospital clients receiving occupational therapy in neurological rehabilitation and in an older adult acute inpatient mental health service | Qualitative | Unclear | Interviews; Grounded theory method | Museum |
| 33 | Beauchet et al. (2018)  Canada | To examine the effect of the MMFA participatory-based art activity on well-being, quality of life and health condition in older community dwellers | Report | 130 participants, aged 65 years or older | Self-Administered Questionnaire, EQ-5D, computer proficiency questionnaire; Statistical analysis | Museum |
| 34 | Beauchet et al. (2020)  Canada | To examine changes in well-being, quality of life and frailty associated with a weekly art-based activity - “Thursday at the Museum” - performed at the Montreal Museum of Fine Arts in community-dwelling older adults | Quantitative | 130 community-dwelling  older adults (mean age 71.6±4.9, 91.5 % female) | Questionnaire; Statistical analysis | Museum |
| 35 | Bengtsson et al. (2015)  Sweden | To investigate whether the Swedish Semantisk  Milj¨obeskrivning (SMB) method can help describe what characterizes an ideal level of pleasantness and other SMB dimensions in the outdoor environment of nursing homes | Quantitative | 26 pensioners (20 women, 6  men; mean age = 72 years); 26  nursing home staff (25 women,  1 man; mean age = 47 years) | SMB tool; Statistical analysis | Garden |
| 36 | Bennington et al. (2016)  United States of America | To identify the therapeutic benefits of utilizing the art museum, in art therapy programs, with older adults | Qualitative | 8 participants, 7 female and 1 male, aged 75 years or older | Artwork, journaling, research observations and session notes; Phenomenological methods | Museum |
| 37 | Brown and Thompson (2020)  United Kingdom | To share museum resources and activities that can support quality of life improvements for older people | Report | N/A | N/A | Museum |
| 38 | Camic et al. (2014)  United Kingdom | To understand the experience of an eight-week art-gallery-based intervention offered at two different galleries for people with mild to moderate dementia and their carers | Mixed-methods | 24 participants  (12 with dementia), aged 58-94 years | Questionnaires and interviews; Statistical analysis, Thematic analysis | Museum (art gallery) |
| 39 | Camic et al. (2019)  United Kingdom | To examine the wellbeing impact of handling museum  artefacts, by testing for differences across domain, time, gender and stages of dementia | Quantitative (quasi-experimental) | 80 participants aged 54-89 years (M = 74.81 years,  SD = 7.6 years) took part in the study (53 males) | Questionnaire; Statistical analysis | Museum |
| 40 | Cann (2017)  United Kingdom | To discuss how the arts and cultural activities are a vital part of a health and care system and have potential to fulfil the theme of active ageing | Commentary piece | N/A | N/A | Museum |
| 20 | Collective Encounters (2020)  United Kingdom | To report findings from a series of free events exploring how the participatory theatre sector was responding to COVID-19 and the effect lockdown and social distancing was having on participants, participatory theatre makers and their practice | Report | N/A | N/A | Theatre (report on the digital but mentions libraries as solutions to challenges) |
| 41 | Culture Health and Wellbeing Alliance (2020)  United Kingdom | To bring to light the scope, breadth and reach of the work that cultural and creative organisations and individuals have been doing since lockdown to reach the most vulnerable | Report | Based on 50 projects from 48 organisations | N/A | Various cultural sectors (Museum) |
| 21 | Cutler (2020)  United Kingdom | To explore how arts organisations have supported the wellbeing of older people during the COVID-19 pandemic, the challenges that they are facing, and what needs to be in place to for sustained support | Report | N/A | N/A | Museum |
| 42 | Duncan (2018)  United Kingdom | To explore the introduction of the Armchair Gallery app,  managed by City Arts, with older people and understanding how creativity and digital technology can be of benefit in later life | Report and evaluation | App used by 228 participants, aged between 50-100 years (203 aged 65+ years) – 167 females; 61 males | Interviews with 10 care staff/volunteers and 3 observations; not clear how data were analysed | Museum; Sculpture park (Garden) |
| 43 | Fancourt et al. (2020)  United Kingdom | To explore associations between community engagement and the risk of dementia incidence over a 12-year period, while controlling for a wide range of social confounders and accounting for the competing risk of death in older adults | Quantitative | Data analysed from 9550 adults aged 50+ from the English Longitudinal Study of Ageing, with baseline from 2004 to 2005 | Questionnaire; Statistical analysis | Museum |
| 44 | Flatt et al. (2015)  United States of America | To describe the subjective experiences of older adults with early-stage Alzheimer’s disease or related cognitive disorders (ADRDs) and their family caregivers who participated in an art museum engagement activity | Qualitative | 10 persons with ADRD (8 were 60 or older and half were female) and 10 family (6=female) caregivers (6 were 60 years or older) | Focus group and survey; Thematic analysis, statistical analysis | Museum |
| 45 | Ford (2012)  Fieldwork conducted in the United States of America | To identify the benefits for individuals with dementia from  participating in highly creative engagement activities | Qualitative | A Winston Churchill Travelling Fellowship study carried out in  six cities across the USA | Data, observations and knowledge were gathered from various access programmes in  museums and galleries and in alternative environments from hospitals, to  day centres and residential care | Museum |
| 46 | Ganga et al. (2017)  United Kingdom | To understand the impact of House of Memories Family Carers Awareness Day and to provide suggestions for the future development of the programme | Mixed-methods | Survey to 50 informal carers (70% aged 50 or older and 91% were female);  8 museum staff interviewed | Pre-post survey – 66 useable responses  8 semi-structured interviews with museum staff  Participant observation of 8 sessions  Social return on investment | Museum |
| 47 | Gould (2013)  United Kingdom | To report findings from A4D’s landmark pilot programme  London Arts Challenge in 2012 (LAC) at arts venues around the capital (a range of activities were explored – we drew data on museums) | Evaluation | 52 people with dementia (42 involved in the evaluation) aged 69-91 years (55%=female)  49 carers (39 evaluated) | Questionnaire (open and closed questions)  Attendance register  Observations | Museum |
| 48 | Goulding (2013)  United Kingdom | To understand how the lives  of older people can be improved by examining their use of contemporary visual art in an art gallery and museum | Qualitative | 43 participants aged 60-92 (17 males, 26 females) | Interviews; no information on data analysis | Museum (art gallery) |
| 49 | Hendriks et al. (2019)  Netherlands | To evaluate the implementation of an interactive museum program for people with dementia and their caregivers and the impact of the program’s implementation on the organization and on the attitudes toward dementia of the museum staff | Mixed-methods | 23 stakeholders interviewed and 176 participants (museum employees) completed the questionnaire | Interviews, Approaches  to Dementia Questionnaire; Content analysis and Statistical analysis | Museum |
| 50 | Hendriks et al. (2019)  Netherlands | To investigate whether responsiveness during museum programs depends on the type of artwork shown and/or characteristics of the person with dementia, such as severity of dementia or specific cognitive impairments | Quantitative (cross-sectional) | 72 participants, 43 female and 29 male, with an average age of 81.33 | Assessment of Art Attributes scale, Interact instrument, Global Deterioration Scale; Statistical analysis | Museum |
| 51 | Howarth et al. (2020)  United Kingdom | To examine how social prescribing and, in particular, nature-based solutions, such  as gardening, can be used as a non-medical asset-based approach by health professionals working in the community to promote health and wellbeing | Mixed-methods | 47 participants aged 30-85 years, although most people are over 60 years of age | Short version of Warwick-Edinburgh  Mental Well-being Scale (before and after visit), focus groups; Statistical analysis, Thematic analysis | Garden |
| 52 | Johnson et al. (2017)  United Kingdom | To compare the impact of two museum-based activities and a social activity on the subjective wellbeing of people with dementia and their caregivers | Quantitative | 66 participants, 36 people with dementia (25 male, 11 female, average age = 74 SD 7.06) and 30 caregivers (4 male, 26 female, average age = 66 SD 9.95) | Visual analog scales, Evaluation questionnaire; Statistical analysis | Museum |
| 53 | Joyce (2005)  United Kingdom | To describe and evaluate an innovative project that brought together the skills and creative vision of a major national museums group in partnership with a housing action trust | More a commentary and lessons learnt rather than a formal evaluation | N/A | N/A | Museum |
| 54 | Liptak et al. (2014)  United States of America | To describe humour and laughter in persons with cognitive impairment and caregivers who were recalling a shared experience in a focus group – all had been part of an art engagement activity at the Andy Warhol Art Museum | Qualitative | 20 participants (10 with cognitive impairment and 10 caregivers), 11 females and 9 males, the majority were 60 years or older | Focus groups (n=4); Content analysis | Museum |
| 55 | Lynch (2019)  United Kingdom | To capture key contributions and learnings from *The Ageing Well: Creative Ageing and the City* symposium | Report | N/A | N/A | Museum |
| 56 | Mmako et al. (2020)  Australia | To explore existing evidence for quality of life impacts of contact with green spaces by people living with dementia in the community | Mixed-methods review | 19 studies | Systematic review; Thematic analysis | Greenspace (Garden) |
| 57 | Museum Development North West (MDNW) (2019)  United Kingdom | To describe an age-friendly standards self-assessment checklist to help assess and monitor an organisation’s progress against the Age-Friendly Standards | Checklist/Report | N/A | N/A | Museum |
| 58 | Roe et al. (2016)  United Kingdom | To identify the benefits and potential impact of an arts for health programme on the  wellbeing of older people from supported living and care home populations, and to evaluate its feasibility | Qualitative | 32 participants, 17 from a supported living facility and care home, 10 care staff and one relative, 4 cultural sector staff | Non-participant observation and group interviews; Content analysis | Museum |
| 59 | Schall et al. (2018)  Germany | To report the results of a randomized wait-list controlled  study on the influence of an arts-based museum intervention on the emotional state, well-being,  and quality of life of people with dementia | Mixed-methods | 88 participants, 44 people with dementia (23=female) and 44 accompanying relatives or informal caregivers | Measures of cognitive and functional status, mental health, quality of life, neuropsychiatric symptoms, wellbeing, observations and written feedback from caregivers observations; Statistical analysis, not clear how more qualitative data were analysed | Museum |
| 60 | Thompson et al. (2020)  United Kingdom | To design and develop the Museum Health and Social Care Service, a resource and training package that reinforces the care and clinical benefits of arts and cultural activities to older people’s health and wellbeing | Consensus and development work | 17 individuals comprising older service users, and staff from a range of health and social care, arts, and museum and archive services | Collating core care needs of older people and then identifying activities to address these and developing advice on their delivery | Museum |
| 61 | Thomson and Chatterjee (2016)  United Kingdom | To evaluate museum interventions for older  adults in differing health care settings using subjective well-being measures | Quantitative  (Pre-post design) | 40 older adults, aged 65-85 years (11=males and 29 females) | Positive Affect and  Negative Affect Schedule, Visual Analogue Scales; Statistical analysis | Museum |
| 62 | Thomson et al. (2018)  United Kingdom | To assess psychological wellbeing in a novel social prescription intervention for older adults called Museums on Prescription and to explore the extent of change over time in six self-rated emotions | Mixed-methods | 115 participants aged 65-94 at risk of social isolation and loneliness (63% = females) | Museum Wellbeing Measure for Older Adults, diaries, interviews; Statistical analysis, not clear how qualitative analysis was undertaken | Museum |
| 63 | Todd et al. (2017)  United Kingdom | To understand how museum programs create opportunities to enhance wellbeing and health, and change experiences of social isolation in older adults | Qualitative | 20 participants aged 64-87 | Interviews, diaries, Grounded theory (including constant comparison, memo taking, open and selective coding) | Museum |
| 64 | Toepoel (2011)  Netherlands | To develop a profile of the social integration behaviour of older people, focusing on the relationship between measures of social integration and satisfaction with life with  different types of cultural activities based on highbrow and lowbrow categories | Quantitative | 3892 participants aged 18-54, 1171 participants aged 55-64, 847 participants aged 65+ | Longitudinal Internet Studies for the Social  Sciences (LISS) Panel, questionnaire on social integration and leisure time; Statistical analysis | Cultural activities (Museum) |
| 65 | Tyack et al. (2017)  United Kingdom | To explore whether art-based interventions can be delivered via a touchscreen tablet device displaying art images | Mixed-methods | 24 participants - 12 pairs of people with dementia (average age = 75) and informal caregivers (average age = 66) | Quality of Life-Alzheimer’s Disease, Visual Analogue Scale, interviews; Statistical analysis, Thematic analysis | Museum |
| 66 | Tymoszuk et al. (2020)  United Kingdom | To investigate whether frequency of receptive arts engagement over 10 years contributes to experienced,  evaluative, and eudaimonic well-being in older adults | Quantitative | 2,767 participants with an average of 62.3 (54% = female) | Used data from the English Longitudinal Study  of Ageing from Wave 2 (2004/2005) to 7 (2014/2005); Statistical analysis | Arts engagement (museum) |
| 67 | Veall et al. (2017)  United Kingdom | To share best practice findings from the Museums on Prescription (2014 – 2017) project, funded by the Arts and Humanities Research Council and carried out by researchers at University College London and Canterbury Christ Church University | Guide/Report | N/A | N/A | Museum |
| 68 | Watts (2015)  United Kingdom | To share findings from six case studies on involving older men in arts organisations, covering recruitment, programming and participation, impact and practical tips | Report based on qualitative case studies | 6 case studies of best practice of engagement with older men; 6 project managers and 31 project participants | Focus groups and interviews; thematic analysis | Museum |
| 69 | Weiner (2014)  Fieldwork undertaken in Australia and the United States of America | To explore how museums can inspire creativity and build exceptional arts programmes for people in later life and how this momentum can be sustained within a museum setting | Report based on participating or talking to stakeholders from organisations in Australia and America as part of a Winston Churchill Travel Fellowship | N/A | N/A | Museum |
| 70 | Whear et al. (2014)  United Kingdom | To examine the impact of gardens and outdoor spaces on the mental and physical well-being of people with dementia who are resident in care homes | Mixed-methods review | 17 studies | Systematic review; thematic analysis | Greenspaces (garden) |
