# Supplementary material for: Tailoring cultural offers to meet the needs of older people during uncertain times: a rapid realist review
Source: BMC Med. 2022 Aug 24;20:260. doi: 10.1186/s12916-022-02464-4 (PMC9398500; doi:10.1186/s12916-022-02464-4)
Supplement: Supplementary file 2 — Additional file 2. A selection of data extracts used to develop Context-Mechanism-Outcome Configurations (CMOCs), which were also informed by our discussions with stakeholders. [file 12916_2022_2464_MOESM2_ESM.docx]

***Additional file 2: A selection of data extracts used to develop CMOCs, which were also informed by our discussions with stakeholders***

| ***Messaging***   - ***CMOC1: When a link worker can provide detailed information about a cultural offer (C), the older person is more likely to understand if it is suitable for them (O) because they can work out what it entails (M).*** - ***CMOC2: When the link worker explains the cultural offer as part of social prescribing in a way that shows how it relates to an individual’s needs (C) because it is regarded as a credible solution (M), the older person is more likely to accept it (O).***   “Be realistic about your offer from the beginning; be clear about what activities you are offering (what, when and where), but don’t overpromise health or wellbeing outcomes.” [68]  “The age sector needs to better understand the local creative offer and ensure that the older people they support know where, when and how to get involved. Equally, the cultural sector needs to shape and communicate their offer more successfully by using the networks that exist to reach those older people who are not already taking part.” [31]  “The arts sector needs to work hard to contribute to Link Workers’ knowledge and experience of the arts and work in partnership (e.g. with NHS) to enhance training.” [55]  “…arts and culture must be helped to escape its marginalised role through a widespread change of hearts and minds. There is a need for advocates of active ageing, including gerontologists, to accord the arts parity of respect and esteem with other, more traditional forms of care and support.” [40]  “Only two residents had been to the gallery before; attending museums or galleries was not the norm for residents or care staﬀ.” [58]  “Another important barrier for engagement in cultural activities by older men is how these activities are branded and presented. Some older men still hold very traditional and separated roles, which includes hobbies and the leisure activities normally associated with members of each gender.” [68]  ***Matching***   - ***CMOC3: When link workers understand the needs and expectations of an older person (C), they are more likely to suggest a suitable cultural offer (O) because they have an understanding of what is acceptable to and needed by that individual (M).*** - ***CMOC4: When a link worker has information of local social prescribing options (C), they can match these to older people's needs and expectations (O) because they have the necessary knowledge (M).***   “Older people are a diverse group in terms of age, gender, race, and socioeconomic class.” [48]  “Attention is needed importantly to those policies such as transport, access and technology which can enable, but currently often disable the realisation of the huge potential of arts and culture.” [40]  “Awareness training on creative activity ideas and beneﬁts to improve health and well-being for the individual with dementia as well as strengthening relationships between staff, carers and loved ones.” [55]  “Aim for your sessions to have a balance between new knowledge and experiences, creative activities and social time.” [67]  “Online support trialled to date has included artists joining Zoom classroom sessions and WhatsApp weekly meetings as well as emails and phone-calls to encourage engagement and dialogue.” [41]  ***Monitoring***   - ***CMOC5: If cultural institutions evaluate the cultural offers they make to older people (C), they can adapt the suitability of the offer (O) because they are aware of the changes needed (M).*** - ***CMOC6: When a link worker asks for feedback from older people attending cultural offers (C) they can assess whether a cultural offer is benefiting someone (M) and changes can be made to the individual’s action plan if required (O).*** - ***CMOC7: When link workers and cultural sector staff collaborate constructively (C), improvements to cultural offers are more likely (O) because their shared knowledge is harnessed (M).***   “Implementation and evaluation plans: At present, the resource is being trialed in local care homes with registered health and social care professionals and care support staff. This will provide the MHSCS team with information about usability of the resource, and training requirements and effective training approaches needed to use the resource.” [60]  “Evaluation is vital in the ongoing development of arts programmes for older adults in museums. It must be recorded and used. In order to understand the results and evaluate how to replicate best practice, museums and galleries need to build-in deliverable evaluation and engage more researchers to widen the reach of their findings.” [69]  “We swiftly learned about using Zoom and conducted an audit of participants’ access to devices and data in our dementia-related projects. We offered multiple test sessions to coach individuals to use the best methods for them – phone, video call, smartphone, laptop, PC…” [21]  “In the present study, pensioners and staff were asked to assess outdoor environments at nursing homes, and it is important to take into account that these 2 groups did not have the same perceptions as residents. The views of the residents themselves, which we were not able to include in the SMB assessments, are crucial for obtaining a richer, deeper, and more detailed understanding of the importance of different environmental qualities in the outdoor environment at nursing homes.” [35]  “Further understanding and appreciation for the immediacy of experience, we would argue, could help public health services, community organisations and charities to better understand which activities to support to further develop…” [39]  “A brief open-ended feedback questionnaire aimed to elicit responses about participants’ experiences was given at the end of the intervention.” [52]  “Building in evaluative measures at the very early planning stages is helpful so that they are an intrinsic and structured part of the whole project … Building on what has been learned and achieved will ideally follow on while the results are still fresh and active in people’s minds.” [53]  “In the testing phase, some of the older people found the visual aspects needed to have  greater contrast in order to navigate and view the app.” [42]  ***Partnerships***   - ***CMOC8: When a cultural organisation is committed to supporting public well-being (C), because staff feel that they are undertaking such work in a facilitative environment (M) they are willing to make changes and take risks (O).*** - ***CMOC9: When older people are consulted about the content of cultural offers (C), something is developed by cultural organisations that is appropriate and acceptable to end users (O) because it has taken into consideration their ideas (M).*** - ***CMOC10: When link workers and cultural sector staff interact (C), it allows for greater understanding and valuing of each party’s contribution to older people’s well-being (M), which promotes a willingness to collaborate (O).***   “…those designing art museum-based interventions for older adults with dementia and caregivers should consider collaborating with clinicians and other trained providers to aid in tailoring programs.” [44]  “We had a team of three staff delivering the programme. We were able to support each other and discuss how each individual participant was responding. This was essential for our own wellbeing and resilience.” [67]  “Given that arts organisations were rarely working online pre-lockdown, and the importance of the digital divide, it is natural that the case studies in this report use a wealth of non-digital methods. One almost forgotten method of communication that has seen a come-back has been the use of the postcard.” [21]  “Collaboration between healthcare providers and museum or arts professionals provides an example of harnessing existing community resources to promote psychosocial wellbeing outside of traditional medical settings.” [52]  “Arts institutions are all very white, and very middle class. We need to think and do more about getting our museums and galleries to take their exhibitions out to where people live. If we are not coming to you, you need to come to us. Not just for a day, tell them that you will be back next week perhaps with something different. Consider coming to neutral spaces - community buildings such as libraries, community centres, schools, leisure centres and coffee shops.” [55]  “Collaborating with link workers will facilitate involvement with local mutual aid groups that could help support those who are self-isolating through the social prescription offer.” [51]  “I knew that to understand why older men were not getting involved in such activities I had to first understand what made those activities, that did appeal, so successful.” [68]  ***Distracting***   - ***CMOC11: When an older person finds the cultural offer stimulating (C), they experience an escape from their problems (O) because they enjoy and are absorbed by the activity (M).*** - ***CMCO12: When the cultural offer engages older people’s senses (C), their enjoyment increases (O) because their mind is elsewhere (M).***   “This routine and repetition contributes to successful programming. Participants and carers feel safe, nurtured and able to focus on the task at hand.” [69]  “The sensory experience associated with [the offering] stimulates imagination, memory, cognition and orientation.” [37]  “83% of PWD (people with dementia)…feel they have learned new skills.” [47]  “‘It takes your mind off your worries – I was in a world of my own.’ J - aged 70 - participant at Mosaic Heritage session, Newport Library, January 2018.” [31]  “Another with a poor prognosis said the session took her mind off the bad news, helping her not to get immersed in it.” [61]  ***Holding***   - ***CMOC13: When the cultural environment is older people friendly (C) they enjoy attending (O) because they feel safe and at ease (M).*** - ***CMOC14: When the cultural offer is delivered professionally and consistently (C), older people feel reassured (O) because they know what to expect (M).***   “To visit a new environment that has no relation or stigma to dementia can be a satisfying and rich experience for the individual and their loved ones. Emphasis is taken off the individual and they become a group unit to learn, discuss, socialise and transform their lives through art history and making.” [45]  “I was treated so well, it made me feel I wasn’t a drain…It was better than the memory clinic because we did something…we were people working together and were treated as equals and not as a person with a memory problem but as two people with something to contribute, and that was a good thing (D-C).” [38]  “The gallery/museum facilitator staff being present to meet the visitors on arrival was agreed as important to welcome them and put them at ease, as was having the buildings accessible for people in wheelchairs or using walkers.” [58]  “Along with considerations for place and length of time, the role of the facilitator as museum expert and as a socially engaging and welcoming person, was seen to be essential.” [63]  “It’s important to remember that the venues you use are an essential part of the audience/participant experience. Inadequate facilities will affect visitors’ ability to enjoy the cultural event and most will not distinguish between the venue and the activity – for them it’s all one experience.” [57]  “For older people, accessibility is most often considered in terms of physical barriers connected to museum architecture and outdoor spaces. When working with older people, it is essential to consider the suitability of stairs, lifts, ramps, handrails and gallery and connecting spaces, rest facilities, retail and eating spaces for visitors with a range of access requirements.” [67]  “One respondent reported that it was ‘sometimes good to remember, but sometimes bad’…A third of interviews included reports of lowered mood; this related to specific images and not the overall art-viewing experience. Two people said some images could be upsetting.” [65]  “We begin looking at best practice by addressing the importance of the caring environment because this appears to underpin the success of arts programmes for older people that have been sustained over many years. Even museums starting out in this type of work felt that they had introduced an ‘enhanced level of care’ for these particular participants.” [69]  “‘The reception area was just outside the workshop room, beneath the BM Great Court at the Clore Education Centre. However, there was no sign at the main entrance or any other part of the museum, just by the registration desk and on the workshop room. Staff on the Museum information desk were not able to inform me about the location of the workshop’ (Observation Note, British Museum).” [46]  ***Connecting***   - ***CMOC15: When the cultural offer provides a social component (C), older people feel less lonely (O) because they have been facilitated to engage in human interactions (M).*** - ***CMOC16: As the cultural offer continues to provide a social component (C), older people can increase their social network (O) because they have been facilitated to develop and maintain new relationships (M).***   “…museum programs supported people taking a chance, and the feeling of being alongside other participants was a catalyst, “If you got stuck on one of the crafts or something, there was somebody from the group to help ” [P4]. These experiences were something shared with others, which led to increased communication, the beginning of new social contacts, and revised appraisals of self and others.” [63]  “…in this study bonding occurred and several participants stayed in touch with one another after the programme; there was evidence of bridging in that participants were especially keen to talk to museum staff who they met on a weekly basis; and short-term linking was noted when participants had the opportunity to talk to artist-educators, students and volunteers encountered for one or two sessions. One participant commented that it brought them into contact with a much wider range of people than they would normally meet, others talked about getting to know people in a ‘different context’ or ‘under different circumstances’.” [62]  “…it allows the public in to do a project within the museum space. I think anyone coming along to this project would get a lot of satisfaction because you are learning a skill but also meeting new people. You’re building up a network of friends.” [68]  “We have so much to tell friends as it’s so different from anything else we do.” [47]  ***Transforming***   - ***CMOC17: When the cultural offer enables older people to experience or learn new things (C), their self-esteem and confidence increase (O) because they are encouraged to try things outside of their comfort zone (M).*** - ***CMOC18: When older people are given the option to take part in a cultural offer in a way that suits their preferences (C), their self-worth is increased (O) because they feel attended to (M).***   “One of the ways the museum built conﬁdence was by providing the space and encouragement to try something new, “This sort of thing, it helps lonely people, helps with conﬁdence as well and I think that's the other thing with being lonely or on your own you haven’t got the conﬁdence to go in on your own” [P12].” [63]  “Feelings related to mastery were also discussed: One person said it felt good to be able to use the app, a person with dementia reported that their confidence in their cognitive abilities was increased. Another pair reported pride in relation to having an app.” [65]  “Patient is talking about the silk screen tote bag art project. Patient: ‘But to see how it gets done and do it ourselves makes you feel good because you did it. Nobody painted this for us. We did it ourselves. You know, and when people look at this. I made this. I made this at the Andy Warhol museum. I’m real tickled about that.” [54]  “Participants acknowledged that not many men engage with cultural activities at later age. Social isolation and lack of self-esteem and confidence were considered to be the main barriers preventing men from engaging in cultural activities.” [68] |
| --- |
